# Computational Analyses of YY1 and Its Target RKIP Reveal Their Diagnostic and Prognostic Roles in Lung Cancer

**DOI:** 10.3390/cancers14040922

**Published:** 2022-02-12

**Authors:** Silvia Vivarelli, Luca Falzone, Caterina Maria Grillo, Benjamin Bonavida, Claudia Crimi, Ignazio La Mantia, Massimo Libra

**Affiliations:** 1 Department of Biomedical and Biotechnological Sciences, University of Catania, 95123 Catania, Italy; silvia.vivarelli7@gmail.com; 2 Epidemiology and Biostatistics Unit, IRCCS Istituto Nazionale Tumori “Fondazione G. Pascale”, 80131 Naples, Italy; 3 Otolaryngology Unit, Department of Medical Sciences, Surgical and Advanced Technologies, University of Catania, 95123 Catania, Italy; grillo.caterinamaria@gmail.com (C.M.G.); ilamantia@unict.it (I.L.M.); 4 Department of Microbiology, Immunology and Molecular Genetics, David Geffen School of Medicine, University of California, Los Angeles, CA 90095, USA; bbonavida@mednet.ucla.edu; 5 Department of Clinical and Experimental Medicine, Section of Respiratory Medicine, A.O.U. “Policlinico-Vittorio Emanuele”, University of Catania, 95123 Catania, Italy; dott.claudiacrimi@gmail.com; 6 Research Centre for Prevention, Diagnosis and Treatment of Cancer, University of Catania, 95123 Catania, Italy

**Keywords:** lung cancer, Yin-Yang 1 (YY1), raf kinase inhibitor protein (RKIP), biomarkers, personalized anti-cancer therapy

## Abstract

**Simple Summary:**

Lung cancer (LC) is the tumor with the highest global mortality rate. Novel personalized therapies are currently being tested (e.g., targeted inhibitors, the immune-checkpoint inhibitors), but they cannot yet prevent the very frequent relapse and generalized metastases observed in a large population of LC patients. Currently, there is an urgent need for novel reliable biomarkers for the prognosis and diagnosis of LC. Through the systematic analysis of multiple deposited expression datasets, this report aims to explore the role of the Yin-Yang 1 (YY1) transcription factor and its target the Raf Kinase Inhibitory Protein (RKIP) in LC. The computational analysis suggested the predictive diagnostic and prognostic roles for both YY1 and RKIP stimulating further studies for proving their implication as novel biomarkers, as well as therapeutically druggable targets in LC.

**Abstract:**

Lung cancer (LC) represents a global threat, being the tumor with the highest mortality rate. Despite the introduction of novel therapies (e.g., targeted inhibitors, immune-checkpoint inhibitors), relapses are still very frequent. Accordingly, there is an urgent need for reliable predictive biomarkers and therapeutically druggable targets. Yin-Yang 1 (YY1) is a transcription factor that may work either as an oncogene or a tumor suppressor, depending on the genotype and the phenotype of the tumor. The Raf Kinase Inhibitory Protein (RKIP), is a tumor suppressor and immune enhancer often found downregulated in the majority of the examined cancers. In the present report, the role of both YY1 and RKIP in LC is thoroughly explored through the analysis of several deposited RNA and protein expression datasets. The computational analyses revealed that YY1 negatively regulates *RKIP* expression in LC, as corroborated by the deposited YY1-ChIP-Seq experiments and validated by their robust negative correlation. Additionally, YY1 expression is significantly higher in LC samples compared to normal matching ones, whereas RKIP expression is lower in LC and high in normal matching tissues. These observed differences, unlike many current biomarkers, bear a diagnostic significance, as proven by the ROC analyses. Finally, the survival data support the notion that both YY1 and RKIP might represent strong prognostic biomarkers. Overall, the reported findings indicate that YY1 and RKIP expression levels may play a role in LC as potential biomarkers and therapeutic targets. However, further studies will be necessary to validate the in silico results.

## 1. Introduction

Lung cancer (LC) represents the second most widely diagnosed cancer, as well as the first cause of death due to a malignancy. An estimate of 2.2 million new LC diagnoses and about 1.8 million deaths for LC were reported during the year 2020 [1]. The most diffused form of LC is non-small cell lung cancer (NSCLC), with a prevalence of about 90%. The remaining 10% of LC cases are represented by small cell lung cancer (SCLC) [2]. NSCLC can be further classified into different subcategories, depending on the histological and molecular features, and including: adenocarcinoma (A), squamous cell carcinoma (S), adeno-squamous carcinoma (AS) and large cell carcinoma (L) [3].

The main risk factor for LC is tobacco consumption, although additional exposures might have a profound effect by triggering LC and other respiratory tumors [4,5]. Currently, the 5-year survival rate for individuals diagnosed with LC varies between 5% and 20%, depending on the tumor stage, as well as the geographical area, underlining the major role played by the individual genetic background [1]. The diagnosis of LC is often made when the disease has already progressed. Recently, novel diagnostic approaches, including low-dose computed tomography (LDCT) for high-risk subjects, are forestalling the time of diagnosis [6,7,8].

Regarding the available treatment options, early-stage NSCLC is preferentially treated with surgery, although such procedure might be followed by a high rate of relapses accompanied by metastasis formation, thus lowering the overall survival probability [9,10]. For advanced LC, different therapies are used, such as platinum-based chemotherapy and/or radiation therapy [11]. Additionally, during the last decade, novel therapeutic approaches have been developed, including the use of targeted therapies against specific kinases or receptors found mutated or overexpressed in specific LC cases. Another novel approach is based on targeting the LC patient’s immune system towards the administration of the immune-checkpoint inhibitors (ICIs) [12,13,14,15,16]. 

The current use of targeted therapies, as well as ICIs, has generally improved the outcome in NSCLC patients. In fact, for these patients the 2-year survival rate increased from 34% to 42% [2]. However, an extended proportion of LC patients does not harbor the standard driver mutations and cannot be treated with available targeted therapies [17]. Furthermore, ICI-treated patients often develop resistance and relapse, as well as life-threatening immune adverse-related events [18]. 

Although several biomarkers have been suggested for the diagnosis and prognosis of LC, in the era of precision medicine, there is still a need to identify “tailored markers” that can be proposed in clinical setting for early diagnosis, prognosis and targeted therapy [19]. Additionally, it would be beneficial to identify diagnostic biomarkers able to optimize the current LDCT protocols, allowing, for example, the identification of the false positives, as well as the stratification of CT-positive subjects [20,21]. Among the challenges currently faced by research on LC biomarker development, there is the need for a deeper knowledge of lung carcinogenesis at both molecular and cellular levels. Moreover, although several novel biomarkers of prognosis have been discovered, there is no robust consensus yet regarding their selection or integrated combination. Finally, the clinical validation of such biomarkers is currently demanding, given its intrinsic resource and time needs [22].

Yin Yang 1 (YY1), a C_2_H_2_-type Zinc finger transcription factor, is very conserved among species. YY1 modulates the expression of about 7% of the human genes, hence affecting many different cellular functions, such as cellular proliferation and survival [23,24]. YY1 can modulate the transcription of target genes directly by binding their regulatory regions [24]. Alternatively, YY1 might interact either with transcriptional co-activators/co-repressors or with chromatin modulating enzymes, thus indirectly regulating the transcription of their targets [25]. 

In cancer, YY1 plays a controversial role [25,26,27]. Regarding LC, YY1 has been seen to work as an oncogene, although the mechanism is still not well defined [24]. It was observed that LC patients with higher levels of YY1 expression develop larger and poorly differentiated tumors with lymph node metastases [28]. Moreover, YY1 overexpression in LC has been reported to promote epithelial-to-mesenchymal transition (EMT) [29,30]. In LC cells and animal models, YY1 was shown to bind various promoters and to induce the expression of several oncogenes, such as the long noncoding RNA-plasmacytoma variant translocation 1, Small Nucleolar RNA Host Gene 16, mitochondrial ribosomal protein L42, Zinc Finger Protein 322 [28,31,32,33]. YY1 also interacts with other co-factors to modulate several targets, such in the case of the HIF-1α, whose interaction promotes the hypoxia-induced stemness of LC [34]. 

Raf kinase inhibitor protein (RKIP), whose gene is also known as *PEBP1*, is involved in the pathogenesis of many cancers, where it has been shown to have pleiotropic functional activities, including the control of cellular proliferation, cell survival, EMT and chemo-radio-immuno-resistance [35]. In the vast majority of cancers, *RKIP* expression has been found to be downregulated or even absent, when compared to its abundance in the adjacent normal tissues [36]. In LC, RKIP has been found to be downregulated at both the transcript and protein levels. Additionally, its lower expression has been associated with higher tumor stage accompanied by lymph nodes metastasis formation [37,38,39,40,41,42]. Yet the pathways behind the regulation of RKIP in LC remain to be fully elucidated [43].

In cancer, YY1 and RKIP are interconnected and able to modulate each other’s expression in an inverse relationship, through several regulatory loops [44]. However, the specific molecular mechanisms involved in LC have not yet been clarified. The present computational investigation aims to better characterize the role of YY1 and RKIP in LC and, more specifically, in NSCLC. The analysis of chromatin binding suggested a direct and negative regulation of YY1 on the *RKIP* gene expression. From the computational assessment of a large collection of LC RNA expression datasets and one protein dataset, it was deduced that YY1 and RKIP were inversely correlated. Together, the two factors might represent a robust two-gene signature with predictive diagnostic, as well as prognostic value in LC, although future validation in LC patients will be needed to corroborate this computational analysis.

## 2. Materials and Methods

### 2.1. Chromatin Binding Prediction and Chromatin-Immunoprecipitation Sequencing (ChIP-Seq) Data Analysis

YY1 DNA-binding prediction towards the *RKIP* regulatory region was examined by using the JASPAR-2020 online matrix tool [45]. The YY1 TF ChIP-Seq experiments on the *RKIP* gene regulatory regions deposited in ENCODE 3 were analyzed using the University of California Santa Cruz (UCSC) Genome Browser. In particular, the integrated regulation from ENCODE tracks was used [46].

### 2.2. Dataset Repositories

The Cancer Genome Atlas (TCGA) lung squamous cell carcinoma (LUSC) and lung adenocarcinoma (LUAD) normalized expression data for *YY1* and *RKIP* transcripts were obtained by using the UCSC Xena online exploration tool [47]. The Gene Expression Omnibus (GEO) database deposited LC microarray datasets (Table 1) and in particular the derived normalized expression data for *YY1* and *RKIP* transcripts were obtained by using the R2 Genomics Analysis and Visualization Platform [48].

YY1 and RKIP protein expression data from Clinical Proteomic Tumor Analysis Consortium (CPTAC) LC dataset data were obtained from the online tool UALCAN analysis [54,55].

*YY1* and *RKIP* gene expressions and cluster distributions within the single LC cell RNA-Seq datasets (Table 2) were analyzed by using the following three interface platforms: the Broad Institute Single Cell Portal, the Cambridge Portal of the Human Cell Atlas (EMBL-EBI) and the user-friendly InteRface tool to Explore Cell Atlas (URECA, Korean Bioinformation Centre KOBIC) [56,57,58]. The cell clustering and t-Distributed Stochastic Neighbor Embedding (t-SNE) methods used for GSE131907 dataset were previously published in detail in [59], and the correlated t-SNE plots were obtained through the use of the URECA visualization interface. Meanwhile, the cell clustering and t-SNE methods for E-MTAB-6653 and E-MTAB-6308 datasets were previously published in [60,61] respectively, and the associated t-SNE plots were obtained by using the EMBL-EBI single-cell portal visualization interface.

### 2.3. Statistical Analyses

Statistical analyses were performed using GraphPad Prism version 9.0 for Windows (GraphPad Software, La Jolla, CA, USA). The results were presented as average ± standard deviation (SD) or as median. Single parameter comparisons between two groups were conducted using two-tailed unpaired Student’s *t*-test. Single parameter comparisons between three or more groups were performed using one-way analysis of variance (ANOVA) with Tukey’s or Dunnett’s multiple comparison test. The *YY1* and *RKIP* correlations in all datasets were evaluated by calculating the Pearson’s correlation coefficient.

The receiver operating characteristic curve (ROC) analyses and subsequent areas under the curve (AUC) calculations were used to predict both *YY1* and *RKIP* diagnostic relevance. In particular, for each specific gene analyzed, normalized expression levels were divided into two classes (i.e., normal vs. tumor, low vs. high expression or low vs. high stage/grade). Subsequently the two groups were analyzed through the ROC function analysis (GraphPad Prism version 9.0). The time-dependent ROC analyses were used to predict both *YY1* and *RKIP* prognostic relevance. For this purpose, a multiple logistic regression analysis was performed to analyze the survival data. The three/four variables considered in each analysis were respectively: the survival outcome, the survival time, *YY1* or/and *RKIP* normalized gene expressions. Through the multiple logistic regression analysis (GraphPad Prism version 9.0), the AUC relative to each ROC curve (hence the gene prognostic relevance) was calculated for each gene, either alone or in combination.

The survival analyses were conducted by using the Kaplan–Meier method. Survival curves were compared through the Log-rank (Mantel-Cox) test for trend. For all statistical analyses, differences were considered significant with *p*-values < 0.05; with * *p* < 0.05; ** *p* < 0.01; *** *p* < 0.001; **** *p* < 0.0001.

## 3. Results

### 3.1. The RKIP Gene Expression Is Directly Repressed by the YY1 Transcription Factor

**Figure 1:**
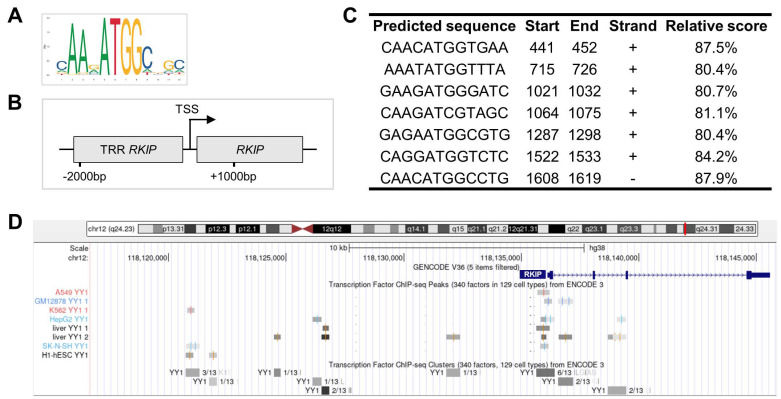
The YY1 TF binds the *RKIP* gene regulatory regions. (**A**) JASPAR prediction matrix of YY1 TF DNA binding sequence. (**B**) Schematic of *RKIP* transcription regulatory region (TRR) and the gene. JASPAR analysis was performed between −2000 bp and +1000 bp around the Transcription Starting Site (TSS) of the *RKIP* gene. (**C**) JASPAR-predicted binding sites of YY1 within the *RKIP* gene regulatory region with location, strand and relative binding score (with 80% cutoff). (**D**) ENCODE 3 YY1-ChIP-Seq-deposited experiments revealed nine YY1-binding clusters, for a total of 23 YY1-binding peaks within eight different experiments (normal and tumor human cells). The genomic region analyzed ranges from 15,000 bp upwards to 5000 bp downwards the TSS of the *RKIP* gene.

To explore the possibility that the *RKIP* gene could be directly regulated by the YY1 TF, an in silico prediction analysis was performed. The JASPAR prediction matrix tool was used to analyze the transcription regulatory region (TRR), ranging from −2000 bp to +1000 bp around the transcription starting site (TSS) of the *RKIP* gene (Figure 1A,B). The analysis showed that YY1 may bind the promoter of *RKIP* at the level of seven different binding sequences, with a relative score included between 80.4% and 87.5% (Figure 1C). To further corroborate the in silico results, deposited YY1-ChIP-Seq data from several experiments performed in cellular specimens were analyzed. As reported in Figure 1D and Supplementary Table S1, the experiments demonstrated the existence of nine different binding clusters located between −15,000 bp and +5000 bp around the *RKIP* TSS, for a total of 23 binding peaks, each one corresponding to the binding of YY1 TF to the DNA of the *RKIP* gene regulatory region. Overall, the results showed in Figure 1 suggested that YY1 TF may directly bind and regulate *RKIP* expression in both normal and transformed human cells.

### 3.2. TCGA Lung Cancer Datasets Analyses Disclose Diagnostic and Prognostic Roles for Both YY1 and RKIP

**Figure 2:**
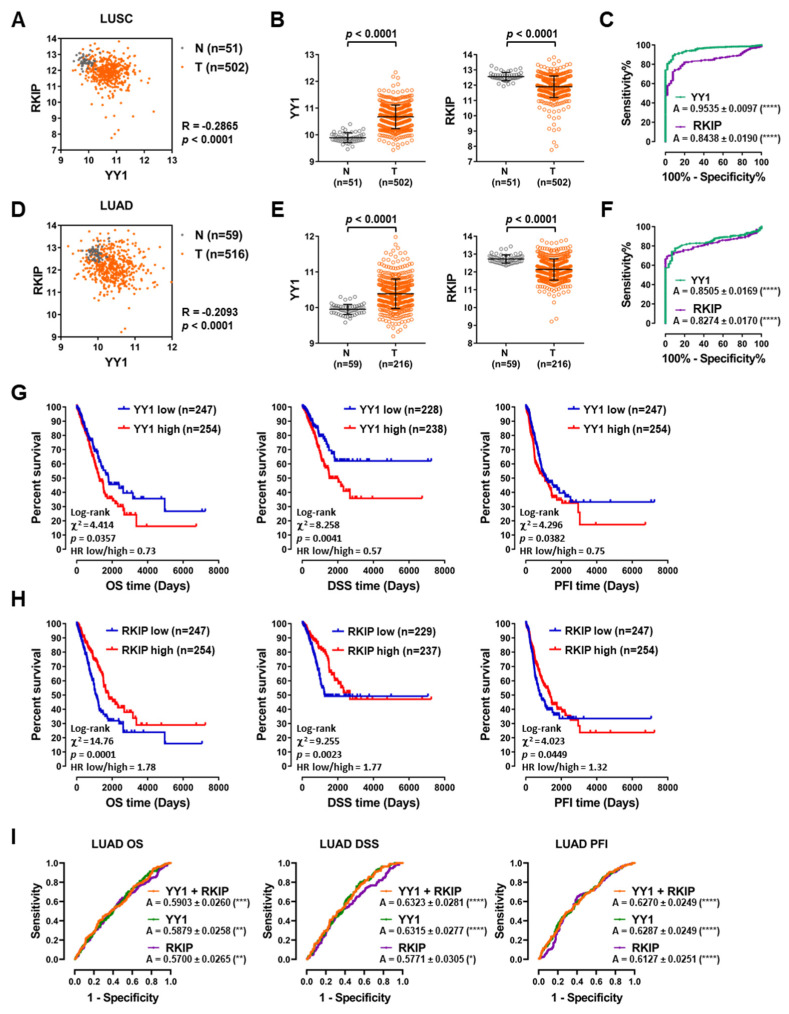
TCGA lung cancer datasets analyses reveal the diagnostic and prognostic values of *YY1* and *RKIP* gene expressions. (**A**) Lung squamous cell carcinoma (LUSC), Pearson’s correlation between *YY1* and *RKIP* gene expression in Tumor (T) and Normal (N) matching tissues. (**B**) LUSC, relative expression of *YY1* (**left**) and *RKIP* (**right**) in N versus T samples. (**C**) LUSC, receiver operating characteristics (ROC) curves and relative areas under the curve (**A**) for *YY1* (green) and *RKIP* (purple) in LUSC samples. (**D**) Lung adenocarcinoma (LUAD), Pearson’s correlation between *YY1* and *RKIP* gene expressions in T and N matching tissues. (**E**) LUAD, relative expression of *YY1* (**left**) and *RKIP* (**right**) in N versus T samples. (**F**) LUAD, ROC curves and relative A for *YY1* (green) and *RKIP* (purple) in LUAD samples. (**G**) LUAD, Overall Survival (OS), Disease-Specific Survival (DSS), Progression-Free Interval (PFI) when patients are stratified in function of *YY1* expression (median expression value is considered to be cutoff). (**H**) LUAD, OS, DSS, PFI when patients are stratified in function of *RKIP* expression (median expression value is considered to be cutoff). (**I**) LUAD, time-dependent ROC analysis and relative A for *YY1* (green), *RKIP* (purple) and *YY1* plus *RKIP* (orange), in correlation with OS (**left**), DSS (middle) and PFI (right). * *p* < 0.05; ** *p* < 0.01; *** *p* < 0.001; **** *p* < 0.0001.

To study the predictive significance of both *YY1* and *RKIP* in LC, two LC TCGA datasets were analyzed: LUSC and LUAD, both including tumor samples and matching normal lung samples. The Pearson’s correlation analysis demonstrated that *YY1* and *RKIP* gene expressions showed a very significant and negative correlation in both LUSC and LUAD samples (−0.2865 and −0.2093, respectively, both with *p* < 0.0001; Figure 2A,D). 

To assess the diagnostic potential of both *YY1* and *RKIP*, the expression levels of both genes were compared between tumor (T) and normal (N) matching samples. Interestingly, in both the LUSC and LUAD datasets, *YY1* was significantly upregulated in T compared to N, whereas *RKIP* was significantly downregulated in T compared to N (Figure 2B,E, all four with *p* < 0.0001). Consistently, the ROC curves in Figure 2C,F showed an AUC of 0.9535 and 0.8505 for *YY1* (both *p* < 0.0001) and of 0.8438 and 0.8274 for *RKIP* (both *p* < 0.0001), in LUSC and LUAD datasets respectively. These high and significant AUC performances suggested that both *YY1* and *RKIP* expression levels can be considered as diagnostic discriminators between the non-transformed and the transformed lung samples.

Interestingly, for the LUAD samples, the subsequent survival analyses and log-rank tests showed that both *YY1* and *RKIP* had a significant prognostic role. In fact, considering the median expression as cutoff value, the Overall Survival (OS), the Disease-Specific Survival (DSS) and the Progression-Free Interval (PFI) were significantly worse in patients with higher *YY1* expression and with lower *RKIP* expression (Figure 2G,H). Correspondingly, the time-dependent ROC curves reported in Figure 2I showed significant AUC performances, for *YY1* and *RKIP* considered either separately or in combination. 

Overall, the results reported in Figure 2 demonstrated that *YY1* and *RKIP* gene expression levels might be robust predictors of diagnosis in LUSC and LUAD patients. Moreover, *YY1* and *RKIP* gene expression levels might be used as strong prognostic predictors in LUAD cases, alone and in combination, as a two-gene prognostic signature.

### 3.3. Lung Cancer GEO Dataset Analyses Confirm Both the Diagnostic and Prognostic Functions of YY1 and RKIP

To further corroborate the diagnostic and prognostic functions of *YY1* and *RKIP* gene expression levels in LC, seven distinct LC Gene Expression Omnibus (GEO) datasets were analyzed (Table 2). Firstly, the Pearson’s correlation analysis demonstrated that *YY1* and *RKIP* gene expression levels are negatively correlated significantly within all the datasets analyzed (Table 2).

**Figure 3:**
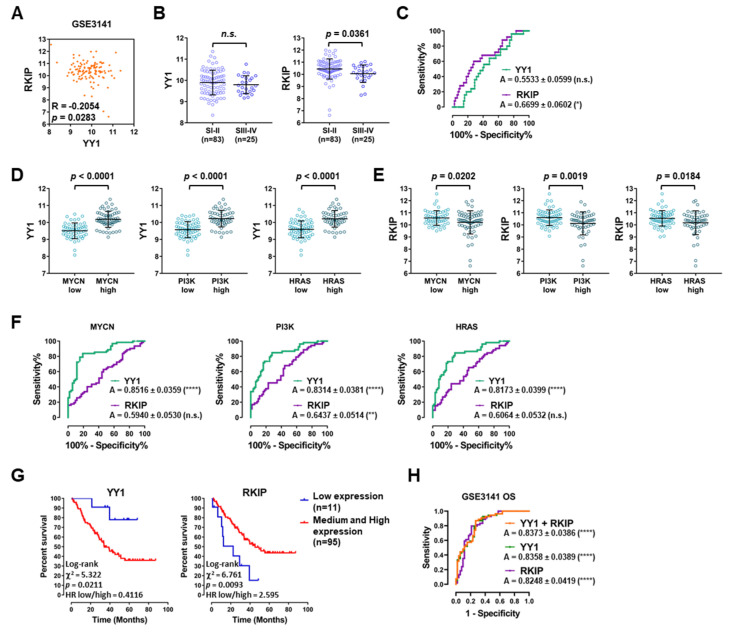
YY1 and *RKIP* are negatively correlated and show a diagnostic and prognostic value in NSCLC GSE3141 Dataset. (**A**) Pearson’s correlation analysis of *YY1* and *RKIP* gene expression (*n* = 114). (**B**) *YY1* and *RKIP* expression in low stage (Stage I, SI and Stage II, SII) versus high stage (Stage III, SIII and Stage IV, SIV) specimens. (**C**) ROC analysis of *YY1* (green) and *RKIP* (purple) in NSCLC samples stratified based on cancer stage. (**D**) *YY1* gene expression in patients stratified based on signature oncogenes expression levels (*MYCN* (**left**), *PI3K* (**middle**), *HRAS* (**right**)). (**E**) *RKIP* expression in patients stratified based on signature genes expression levels (*MYCN (***left**), *PI3K* (**middle**), *HRAS* (**right**)). (**F**) ROC analysis of *YY1* (green) and *RKIP* (purple) in NSCLC samples stratified based on *MYCN* (**left**), *PI3K* (**middle**), *HRAS* (**right**) gene expression level. (**G**) Survival analysis when patients are stratified in function of their OS in relation to *YY1* (**left**) and *RKIP* (**right**) expression (90 percentile value is considered the cutoff). (**H**) Time-dependent ROC analysis and relative A for *YY1* (green), *RKIP* (purple) and *YY1* plus *RKIP* (orange), in correlation with OS. * *p* < 0.05; ** *p* < 0.01; **** *p* < 0.0001.

In particular, the GSE3141 dataset composed of 114 NSCLC samples showed a Pearson’s correlation value of −0.2054 (*p* = 0.0283; Figure 3A). When the samples were stratified based on their staging, while *YY1* showed no significant difference, *RKIP* was lower in higher stage samples (Stages III and IV) compared to lower ones (Stages I and II; Figure 3B). Correspondingly, the ROC analysis showed a significant AUC performance for *RKIP* expression in function of the tumor stage (0.6699, with *p* = 0.0153; Figure 3C).

The GSE3141 NSCLC samples were further stratified based on their molecular signature, corresponding to the expression levels of three of the main driver oncogenes in LC: *MYCN*, *PI3K* and *HRAS* (each divided in low and high expression sample groups). Pivotally, in correlation to the high expression level of each oncogene, *YY1* was found significantly upregulated, whereas *RKIP* was found significantly downregulated (Figure 3D,E respectively). Accordingly, the ROC analyses for *MYCN*, *PI3K* and *HRAS* showed high and significant AUC performances for *YY1*, whereas the AUC performance was significant for *RKIP* gene only in correlation to *PI3K* expression levels (Figure 3F).

Additionally, for GSE3141 it was possible to analyze both *YY1* and *RKIP* gene expression levels in correlation with the OS of the NSCLC patients (with 90% percentile considered as cutoff value). Interestingly, the log-rank test results demonstrated that both *YY1* and *RKIP* had a significant prognostic role. Noteworthy, the OS was significantly worse in patients with higher *YY1* expression and lower *RKIP* expression (Figure 3G). Coherently, the time-dependent ROC curves reported in Figure 3G showed high and significant AUC performances, for both *YY1* and *RKIP* considered separately, as well as in combination, as a two-gene signature.

**Figure 4:**
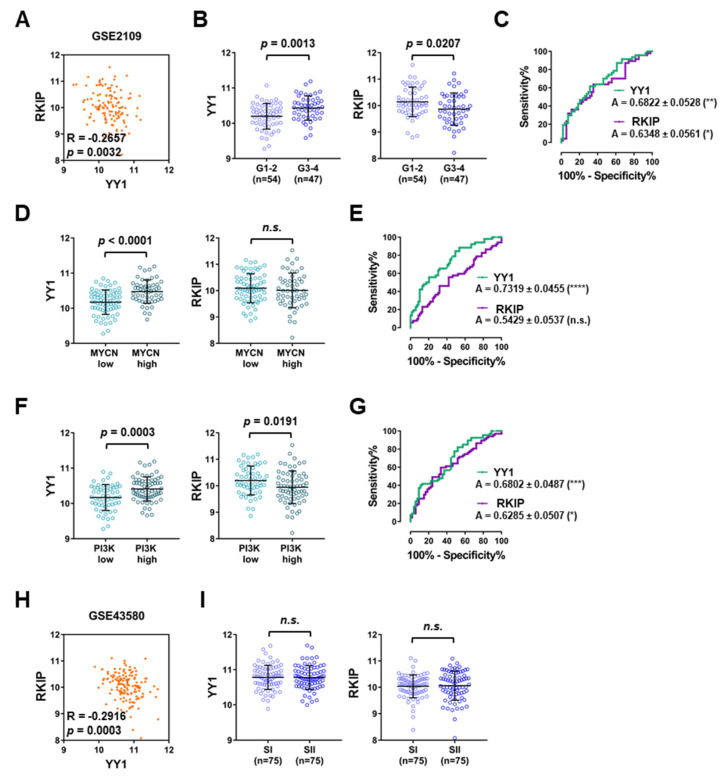
YY1 and *RKIP* are negatively correlated and show a diagnostic and prognostic value in NSCLC Datasets. (**A**) GSE2109, Pearson’s correlation analysis of *YY1* and *RKIP* gene expression (*n* = 121). (**B**) GSE2109, *YY1* and *RKIP* expression in low grade (Grade 1, G1 and Grade 2, G2) versus high grade (Grade 3, G3 and Grade 4, G4) specimens. (**C**) GSE2109, ROC analysis of *YY1* (green) and *RKIP* (purple) in NSCLC samples stratified based on Grade. (**D**) GSE2109, *YY1* (**left**) and *RKIP* (**right**) expression in patients stratified based on signature oncogene *MYCN* expression levels. (**E**) GSE2109, ROC analysis of *YY1* (green) and *RKIP* (purple) in NSCLC samples stratified based on *MYCN* expression. (**F**) GSE2109, *YY1* (**left**) and *RKIP* (**right**) expression in patients stratified based on signature oncogene *PI3K* expression levels. (**G**) GSE2109, ROC analysis of *YY1* (green) and *RKIP* (purple) in NSCLC samples stratified based on *PI3K* expression. (**H**) GSE43580, Pearson’s correlation analysis of *YY1* and *RKIP* gene expression (*n* = 150). (**I**) *YY1* and *RKIP* expression in low stage (SI and SII) versus high stage (SIII and SIV) specimens. * *p* < 0.05; ** *p* < 0.01; *** *p* < 0.001; **** *p* < 0.0001.

The GSE2109 dataset, consisting of 121 NSCLC patients was also analyzed. Coherently with the previous datasets, *YY1* and *RKIP* were found significantly and negatively correlated (Pearson’s correlation −0.2657, *p* = 0.0032; Figure 4A). Upon stratification of the samples based on tumor grade, *YY1* was found significantly upregulated in higher grade samples (Grades 3 and 4, G3–4) compared to lower grade ones (Grades 1 and 2, G1–2), whereas *RKIP* was significantly downregulated (*p* = 0.0013 and *p* = 0.0207, respectively; Figure 4B). Consistently, the ROC analysis showed a significant AUC for both *YY1* and *RKIP* (0.6822 and 0.6348, respectively; Figure 4C). Contrarywise, both *YY1* and *RKIP* expression levels were not significantly different when the samples were stratified based on their stage (Supplementary Figure S1).

The GSE2019 NSCLC samples were further stratified based on their molecular signatures for the expression of two driver oncogenes: *MYCN* and *PI3K* (each divided into low and high expression sample groups). Interestingly, *YY1* was significantly upregulated in samples expressing high levels of *MYCN*, as well as *PI3K*, whereas *RKIP* was downregulated with the higher expression of *PI3K*, and not *MYCN* (Figure 4D,F). The ROC curve analyses reported in Figure 4E,G further demonstrated that the AUC performance was high and significant for *YY1*, with values of 0.7319 and 0.6802 for *MYCN* and *PI3K*, respectively. Meanwhile, for *RKIP* the AUC was significant only in correlation with *PI3K* expression levels (with a value of 0.6285).

Finally, a third dataset, GSE43580, composed of 150 NSCLC patients, was analyzed. In this dataset *YY1* and *RKIP* were negatively correlated in a significant fashion (Pearson’s correlation value of −0.2916, with *p* = 0.0003, Figure 4H). When the samples were stratified based on their stage, neither *YY1* nor *RKIP* were significantly affected (Figure 4I).

Overall, the results shown in Figure 3 and Figure 4 demonstrated that in all three NSCLC datasets analyzed, both *YY1* and *RKIP* gene expression levels were negatively correlated. Moreover, *YY1* was significantly overexpressed in NSCLC in association with the high expression levels of the driver oncogenes *MYCN*, *PI3K* and *HRAS*, whereas *RKIP* was downregulated. Furthermore, the ROC analyses revealed that the assessment of *YY1* levels might be predictive for a certain NSCLC molecular subtype, as high *YY1* expression was correlated with a more aggressive phenotype characterized by the higher expressions of *MYCN*, *PI3K* and *HRAS* signature oncogenes. Interestingly, low *RKIP* expression was correlated with a more aggressive phenotype characterized selectively by the higher expression of *PI3K* gene alone. Finally, the OS analysis performed for the GSE3141 dataset, showed that both *YY1* and *RKIP* might have a significant prognostic role in NSCLC patients. This was further corroborated by the time-dependent ROC analysis which evidenced a high and significant AUC performance for both *YY1* and *RKIP*, either alone or in combination as a two-gene signature. Hence, in agreement with the TCGA LUAD dataset (Figure 2), also in GSE3141 the prognosis was significantly worse in patients with higher *YY1* expression and lower *RKIP* expression.

### 3.4. NSCLC Mixed Dataset Analyses Suggest That YY1 and RKIP Expression Assessments Have a Robust PreDIctive Power

**Figure 5:**
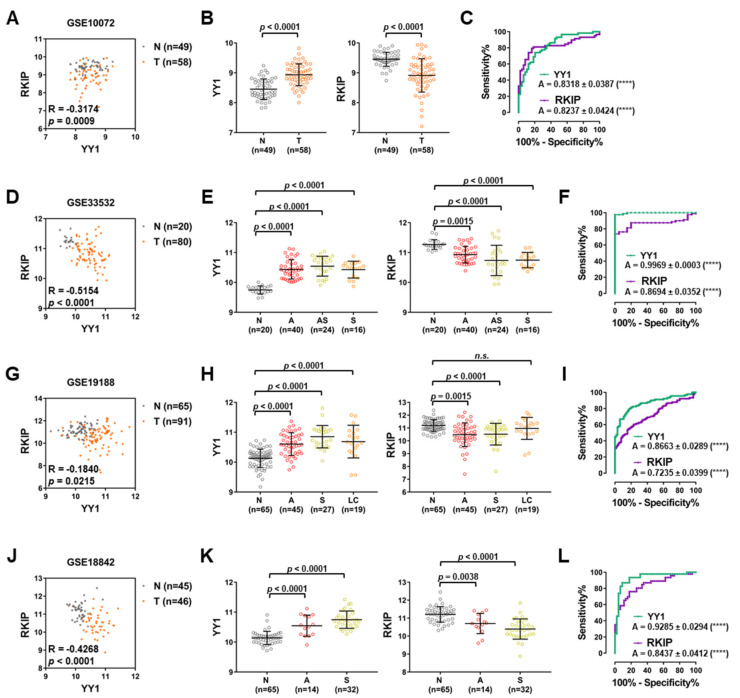
Mixed lung cancer and matching normal samples GEO Datasets analyses reveal a robust diagnostic value for YY1 and *RKIP*. (**A**) GSE10072 Pearson correlation analysis of YY1 and *RKIP* expression in lung Tumor (T) and Normal (N) matching samples. (**B**) GSE10072, relative expression of YY1 (**left**) and *RKIP* (**right**) in N versus T samples. (**C**) ROC analysis of YY1 (green) and *RKIP* (purple) in GSE10072. (**D**) GSE33532 Pearson’s correlation analysis of YY1 and *RKIP* expression in lung T and N matching samples. (**E**) GSE33532, relative expression of YY1 (**left**) and *RKIP* (**right**) in N versus T samples; T samples are divided in groups depending on the histological type: A, Adenous, AS, Adenous-squamous, S, Squamous. (**F**) ROC analysis of YY1 (green) and *RKIP* (purple) in GSE33532. (**G**) GSE19188 Pearson correlation analysis of YY1 and *RKIP* expression in lung T and N matching samples. (**H**) GSE19188, relative expression of YY1 (**left**) and *RKIP* (**right**) in N versus T samples; T samples are divided in groups depending on the histological type: A, S, LC, Large Cell. (**I**) ROC analysis of YY1 (green) and *RKIP* (purple) in GSE19188. (**J**) GSE18842 Pearson correlation analysis of YY1 and *RKIP* expression in lung T and N matching samples. (**K**) GSE18842, relative expression of YY1 (**left**) and *RKIP* (**right**) in N versus T samples; T samples are divided in groups depending on the histological type: A, S. (**L**) ROC analysis of YY1 (green) and *RKIP* (purple) in GSE18842. **** *p* < 0.0001.

Four NSCLC GEO datasets with mixed samples, T and lung matching N, were further analyzed. All four datasets showed a significantly negative correlation between *YY1* and *RKIP* expressions (Figure 5A,D,G,J). In particular, in GSE10072, composed of 58 T and 49 N matching samples, *YY1* was significantly upregulated in T versus N samples, whereas *RKIP* was significantly downregulated (Figure 5B). To assess the diagnostic significance, a ROC analysis was performed, and, as shown in Figure 5C, both *YY1* and *RKIP* relative AUC were high and significant (respectively 0.8318 and 0.8237, both with *p* < 0.0001).

Furthermore, in GSE33532 composed of 80 T samples and 20 matching N samples, the correlation observed between *YY1* and *RKIP* gene expressions was significantly negative (Pearson’s correlation −0.5154 with *p* < 0.0001; Figure 5D). The 80 T samples were subsequently subdivided based on the LC type in adenocarcinomas (A), squamous cell carcinomas (S) and adeno-squamous carcinomas (AS). Interestingly, all T types were significantly different from N matching samples, in terms of both *YY1* and *RKIP* gene expressions. In particular, *YY1* was significantly highly expressed in A, AS and S samples compared to N, while *RKIP* was significantly lowly expressed (Figure 5E). The ROC curve analysis demonstrated that both *YY1* and *RKIP* relative AUC were high and significant (respectively 0.9969 and 0.8694, both with *p* < 0.0001; Figure 5F).

Additionally, GSE19188, composed of 91 T samples and 65 matching N samples showed a significantly negative Pearson’s correlation between *YY1* and *RKIP* gene expressions (correlation value of −0.1840 with *p* = 0.0215; Figure 5G). In this dataset, NSCLC samples could be subdivided based on their specific subtypes in A, S and large cell carcinoma (LC). Importantly, all T samples showed a significant upregulation of *YY1* gene expression in comparison with N matching samples, whereas *RKIP* was significantly downregulated (Figure 5H). Consistently, the ROC analysis demonstrated a diagnostic significance for both *YY1* and *RKIP* genes, with high significant AUC (respectively 0.8663 and 0.7235, both with *p* < 0.0001; Figure 5I).

Finally, for the GSE18842 dataset, made of 46 T samples and 45 N matching samples showed a negative Pearson’s correlation between *YY1* and *RKIP* gene expressions (−0.4268 with *p* < 0.0001; Figure 5J). In agreement with the above-described datasets, when T samples were stratified based on their type in A and S, both T subtypes showed a higher and significant expression of *YY1* compared to matching N whereas *RKIP* expression was significantly lower. Consistently, the ROC curve analysis demonstrated the diagnostic significance of *YY1* and *RKIP* gene expression levels in this dataset as well, with AUC of 0.9285 and 0.8437, respectively (both with *p* < 0.0001; Figure 5L).

Taken together, the results shown in Figure 5 demonstrated that within all four datasets composed of NSCLC samples and normal matching lung N samples, both *YY1* and *RKIP* can be suggested as robust diagnostic discriminators.

**Figure 6:**
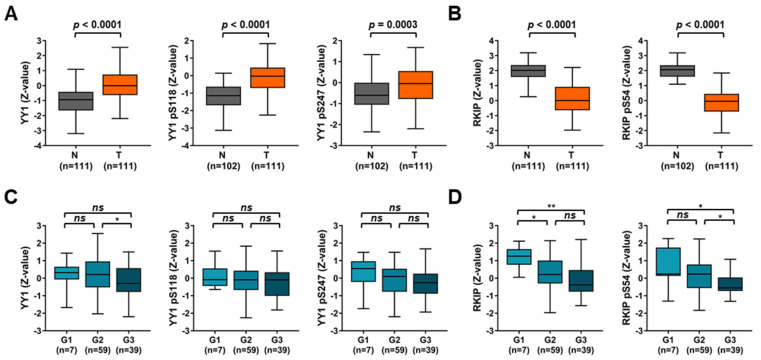
CPTAC analysis of YY1 and *RKIP* protein expression reveals a differential expression in NSCLC versus normal matching samples. (**A**) YY1 (left), phospho-pS118 YY1 (middle) and phospho-pS247 YY1 (right) protein expression in Normal (N) versus Tumor (T) samples. (**B**) RKIP (left), phospho-pS54 RKIP (right) protein expression in N versus T samples. (**C**) YY1 (left), phospho-pS118 YY1 (middle) and phospho-pS247 YY1 (right) protein expression in T samples divided depending on grade. (**D**) RKIP (left), phospho-pS54 RKIP (right) protein expression in T samples divided depending on grade. * *p* < 0.05, ** *p* < 0.001.

To further corroborate the gene expression results, a protein database was analyzed. Specifically, an NSCLC database of 111 T samples and 111 matching N samples (108 for the phosphorylation analysis). Intriguingly, YY1 total protein, as well as phospho-YY1 forms, p-Serine118 (pS118) and p-Serine247 (pS247), showed a significantly higher expression in T samples compare to N ones (Figure 6A). In contrast, both total RKIP and p-serine54 (pS54) were significantly lower in T samples compared to N ones (Figure 6B). In particular, when T samples were grouped based on their grade (G), only RKIP and pS54-RKIP levels were significantly reduced in higher grade samples (G2 and G3) compared to G1, while the results for YY1 were found not significant (Figure 6C,D). Although this result might be proven in additional larger cohorts, it confirmed that the higher levels of YY1 and the lower levels RKIP protein in T samples compared to matching N samples reflected what it was observed at the gene levels. Consequently, YY1 and RKIP protein detection, total and phosphorylated, might also possess diagnostic value.

### 3.5. Single-Cell RNA-Seq Lung Cancer Dataset Analyses Reveal That Both YY1 and RKIP Gene Expressions Are Cell Type-Dependent

**Figure 7:**
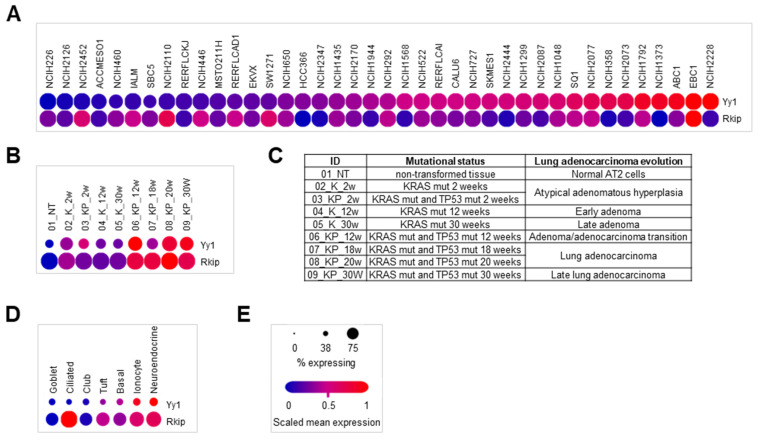
Single cell RNA-sequencing analyses of deposited datasets mouse and human lung cancer and normal tissues reveal an heterogenous and cell-specific expression of *YY1* and *RKIP*. (**A**) SCP542 (53,513 cells). Single cells deriving from 40 different standard human LC cell lines. Analysis of *YY1* and *RKIP* relative gene expression. (**B**) GSE152607, expression profiling of *Yy1* and *Rkip* by high throughput sequencing in single cells (3891 cells) from mouse adenocarcinoma cells evolved through seven sequential stages. (**C**) Summary of the features of each single cellular group in terms of cancer stage. (**D**) GSE103354, expression profiling of *Yy1* and *Rkip* by high throughput sequencing in single cells (7193 cells) of mouse airway epithelial cells. (**E**) Single cell profiling expression legend.

To further explore the expression of *YY1* and *RKIP* genes in LC, novel deposited single-cell RNA-Seq datasets were analyzed. In particular, the Broad Institute’s single cell portal SCP542 study (53,513 cells) was analyzed with respect to 40 different and widely used LC cell lines. Although showing different expression levels depending on the considered cell line, *YY1* and *RKIP* levels were inversely correlated in almost all the cell lines studied (Figure 7A). 

Moreover, a mouse dataset of single LC cells, GSE152607 (3891 cells) was examined. The genetic mouse LC model used showed the evolution of the lung from normal non-transformed (NT), to hyperplastic, to finally adenomatous tissue. The transformation timeline is 30 weeks, and it is driven by the mutation of both *KRAS* and *TP53* oncogenes, either alone or combined. When the single cells were analyzed and pooled based on their adenocarcinoma stage, interestingly both *Yy1* and *Rkip* genes increased in their expression, and *Rkip* was expressed in a higher percentage of cells compared to *Yy1* (Figure 7B,C). While *Yy1* reflects what reported above in human LC datasets, the differences concerning *Rkip* expression compared to what found in human be due to interspecies diversity. In addition, a normal lung mouse tissue single-cell dataset, GSE103354 (7193 cells) was analyzed. There, the expression of *Yy1* was always reduced and present in a reduced percentage of the overall cells, compared to *Rkip*, which is what expected for normal non-transformed lung tissues (Figure 7D). Overall, the results reported in Figure 7 suggest that both *YY1* and *RKIP* expression, although inversely correlated, depends on the specific tumor features.

## 4. Discussion

LC remains the most incurable tumor. Given its heterogeneous mutational landscape, the latter available therapies are biomarker driven [20]. The targeted therapies are tailored to the patient based on the specific genetic background of the tumor (e.g., mutation or altered expression of *EGFR*, *KRAS*, *BRAF*, *PIK3CA*, *PTEN*, *HER2*, or gene fusion of *ALK*, *ROS1*, *RET*) [65]. Additionally, both the levels of PD-L1 tumor expression and the tumor mutational burden might be indicators of a favorable response to the ICIs. Regarding the diagnosis, the currently used serum biomarkers are few (e.g., prolactin, CEA, CYFR21) [66,67]. Overall, only the 30% of LC are diagnosed early, whereas in the remaining 70% of cases the diagnosis is made when the tumor is already at an advanced stage. Moreover, upon therapy, often patients develop resistance to the treatment and relapse of the disease. Therefore, second- and third-line treatments are necessary. For such reasons, the overall prognosis remains poor and the mortality rate very high. 

In this context, it is important to seek novel robust biomarkers of diagnosis and prognosis of LC, to help to stratify the patients and to guide therapy choices. The aim of this computational study is to assess the role of two important cancer factors, YY1 and RKIP as novel predictive biomarkers in LC. YY1 has been known to work as an oncogene in cancer and one of its main the transcriptional targets in LC may be the immune-enhancer and tumor-suppressor RKIP. In several cancers, an inverse correlation between the pro-tumorigenic YY1 and the anti-tumorigenic RKIP has been observed [68].

*YY1* gene expression is positively regulated by NF-κB transcription factor [69]. In turn, NF-κB is inhibited by RKIP in association with NIK and TAK1 [70]. Both NF-κB and YY1 may directly induce the expression of the oncogenic transcription factor *Snail* [71,72]. Snail is a known direct inhibitor of *RKIP* gene expression [73]. In cancer cells, YY1 might inhibit *RKIP* gene expression indirectly, through *Snail* positive regulation. In turn, Snail might inhibit *YY1* gene expression [74]. 

In addition to this indirect regulatory loop, it has also been observed that YY1 might directly inhibit the expression of *RKIP* in several cellular cancer models, as also supported by some unpublished observations [44,68,75]. In this study, we explored the direct interaction between YY1 and *RKIP*, herein supported by several results. First of all, it was shown that YY1 may bind *RKIP* promoter, as evidenced from the JASPAR predictive analysis and consequently confirmed by YY1-ChIP-Seq results. The latter demonstrated that YY1 is able to bind *RKIP* promoter and enhancer in several different cellular models, both normal and cancerous.

Importantly, two TCGA LC RNA expression datasets (LUSC and LUAD) and seven GEO-deposited LC RNA-array datasets were systematically analyzed. These analyses consistently demonstrated the existence of a significant negative correlation occurring between *YY1* and *RKIP* gene expressions within all the considered datasets (Table 1).

Among these datasets, the ones bearing mixed samples (both TCGA and GEO-deposited), T and N matching, demonstrated that *YY1* was highly expressed in T compared to N, whereas *RKIP* was lowly expressed in T samples. For all the mixed datasets, the ROC analysis showed a high and significant AUC, meaning that the assessment of both *YY1* and *RKIP* levels of expression might be used as novel predictive diagnostic biomarkers in NSCLC (Figure 3 and Figure 5). Additionally, all the mixed datasets containing samples obtained from different NSCLC subtypes, showed to have a significantly high *YY1* and significantly low *RKIP* expressions compared to N, but without any difference in the expression within the various tumor subtypes. This suggests that the diagnostic value is independent from the specific subtype of NSCLC (Figure 5).

The protein dataset analysis further demonstrated that not only *YY1* and *RKIP* genes, but also YY1 and RKIP proteins (both total and phosphorylated forms) are differentially expressed in N versus T samples, confirming the trend observed in the gene expression datasets. In particular, YY1 and p-YY1 were highly expressed whereas RKIP and p-RKIP were lowly expressed in NSCLC samples compared to the N counterparts. This result indicates that not only the transcript levels but also the protein abundance of both YY1 and RKIP might be used as diagnostic indicator of the occurring LC transformation (Figure 6). In addition, it was performed a search for the immunohistochemistry (IHC) NSCLC samples deposited in the Human Protein Atlas (HPA) databank. Although the reduced number of HPA-deposited samples (12 Tumor and 3 Normal) does not allow to draw any statistically relevant conclusion, the images show a similar trend for both YY1 and RKIP protein levels (Supplementary Figure S2) [76,77]. Larger LC cohort IHC studies are needed in the future.

In contrast, *YY1* and *RKIP* expression levels did not demonstrate to be univocally predictive of a specific stage or grade of NSCLC within all the datasets analyzed. Only for one dataset was a positive correlation with LC grade for *YY1* and a negative correlation for *RKIP* observed (Figure 4B,C). Meanwhile, in the other two datasets, only *RKIP* was revealed to be negatively correlated with LC stage (Figure 3B) or grade (Figure 6D). These results might support a functional role played by both *YY1* and *RKIP* at the earlier stages of lung cellular transformation, from normal to malignant (Figure 2 and Figure 5). 

By looking at specific stages of lung adenocarcinoma evolution in a genetic mouse model, single-RNA seq data showed that *Yy1* had an increased expression from NT to late adenoma stages. This might support the role of YY1 as main oncogene in both human and mouse. In contrast with human data, in mouse, *Rkip* expression was found increased during the lung cancer evolution. This latter observation might be linked to interspecies diversity in terms of regulatory networks occurring between *Yy1* and *Rkip*, and in particular with respect to the specific function of *Rkip* as tumor suppressor gene in mouse (Figure 7).

Two datasets, GSE3141 and GSE2109, contained samples that could be stratified based on the levels of expression of different driver genes: *MYCN*, *KRAS* and *PI3K*. Interestingly, *YY1* was upregulated concomitantly with the higher expression of all the three considered oncogenes, whereas *RKIP* was down-expressed. Furthermore, the ROC curve analysis showed a diagnostic significance for *YY1* for all the three oncogenes taken into analysis, whereas *RKIP* diagnostic significance was limited only to *PI3K*. Hence, in LC, *YY1* might be considered a driver oncogene involved in the cellular transformation process, while *RKIP* might be considered a tumor-suppressor (Figure 3 and Figure 4).

For the TCGA LUAD dataset, as well as for the GSE3141, *YY1* high expression and *RKIP* low expression were both correlated with a worse survival outcome. These results, further supported by the significant AUC performances of the time-dependent ROC curves (Figure 2I and Figure 3H), strongly indicate a prognostic role for both *YY1* and *RKIP* in NSCLC and, more specifically, in the lung adenocarcinoma subtype. This means that monitoring *YY1* and *RKIP* gene expressions—both alone and in combination as a two-gene signature—might really help to tackle the prognostic window, as well as to match the specific genetic features of each single lung adenocarcinoma patient, with the final goal of suggesting a personalized therapeutic protocol.

Additionally, the expressions of *YY1* and *RKIP* were analyzed at a single cell level (Supplementary Figures S3 and S4). The t-SNE plots showed the tumor-derived cells divided in clusters depending on their cellular nature. While *YY1* was highly expressed specifically in epithelial LC cells, *RKIP* was highly expressed only within a smaller subcluster of epithelial LC cells and also in myeloid cells, mast cells and fibroblasts (Supplementary Figure S3A). Within the clusters of cells from non-transformed matching tissues, *YY1* expression was almost non-detectable in all the clusters, whereas *RKIP* was highly expressed, in particular in the epithelial cells, mast cells, B cells and in a subgroup of myeloid cells (Supplementary Figure S3B). Overall, the single cell RNA-seq data demonstrated that both *YY1* and *RKIP* are heterogeneously expressed within separate subclusters of LC cells and non-transformed matching cells. This further supports the idea that YY1 oncoprotein might directly inhibit *RKIP* gene expression selectively within the lung tumor cells of epithelial origin. In the future, both *YY1* and *RKIP* molecular function must be further studied at the cellular resolution in larger cohorts of LC patients.

## 5. Conclusions

Overall, in light of personalized therapy, this large-scale dataset analysis suggests a potential role of both YY1 and RKIP as novel two-signature biomarkers for LC, with a diagnostic as well as a prognostic significance. This computational analysis further indicates that *YY1* plays as oncogene in LC, whereas *RKIP* as tumor suppressor. The identification of selective YY1 inhibitors and potent RKIP inducers is currently a goal to be pursued in precision oncology [78]. In the future, it will be important to characterize the mechanisms underneath the selective modulation of both YY1 and RKIP expression in NSCLC. In particular, the inhibition of YY1 in transformed cells might have the effect of contrasting the cellular transformation through the modulation of its several targets, including RKIP, which is an inhibitor of the cellular proliferation, the EMT and the metastasis formation.

## Figures and Tables

**Table 1:** Gene Expression Omnibus (GEO) database-deposited LC microarray datasets.

GEO ID	Contributors	Platform	Tumor Samples	Matching Non-Tumor Samples	Pearson Correlation	*p*-Value	Reference
GSE3141	Bild AH et al.	Affymetrix HG-U133 2.0	114	0	−0.2054	0.0283	[49]
GSE2109	n.a.	Affymetrix HG-U133 2.0	121	0	−0.2657	0.0032	n.a.
GSE43580	Peitsch MC et al.	Affymetrix HG-U133 2.0	150	0	−0.2916	0.0003	[50]
GSE10072	Jen J et al.	Affymetrix HG-U133A	58	49	−0.3174	0.0009	[51]
GSE33532	Muley T et al.	Affymetrix HG-U133 2.0	80	20	−0.5154	<0.0001	n.a.
GSE19188	Hou J et al.	Affymetrix HG-U133 2.0	91	65	−0.1840	0.0215	[52]
GSE18842	Farez-Vidal ME et al.	Affymetrix HG-U133 2.0	46	45	−0.4268	<0.0001	[53]

n.a., not associated.

**Table 2:** Single RNA-Seq datasets of LC cells.

Study ID	Technology	Number of Cells	Analysis Portal	Reference
SCP542	Droplet-based scRNA-seq (H sapiens)	53,513	Single cell Portal (Broad Institute)	[62]
GSE152607	Illumina NextSeq 500 (M musculus)	3891	Single cell Portal (Broad Institute)	[63]
GSE103354	Illumina NextSeq 500 (M musculus)	7193	Single cell Portal (Broad Institute)	[64]
GSE131907	Illumina HiSeq 2500 (H sapiens)	45,149; 42,995	URECA (Kobic Center)	[59]
E-MTAB-6653	Droplet-based scRNA-seq (H sapiens)	33,208	Single cell expression atlas (EMBL-EBI)	[60]
E-MTAB-6308	Droplet-based scRNA-seq (H sapiens)	56,771	Single cell expression atlas (EMBL-EBI)	[61]

## Data Availability

The raw data computationally analyzed in this study were deposited in the Zenodo platform (https://doi.org/10.5281/zenodo.5674736; Last accessed on: 11 November 2021) and are also available from the corresponding authors under reasonable request.

## Supplementary Materials

The following are available online at https://www.mdpi.com/article/10.3390/cancers14040922/s1, Table S1: YY1-ChIP sequencing results (from 15,000 bp upwards to 5000 bp downwards the TSS of *RKIP* gene); Figure S1: GSE2109, YY1 (left) and RKIP (right) expression in low stage (SI and SII) versus high stage (SIII and SIV) specimens; Figure S2. Immunohistochemistry (IHC) analysis of YY1 and RKIP protein expression in Lung Cancer and Normal tissue from the Human Protein Atlas (HPA). Figure S3: Single cell sequencing analyses of GSE131907 single cell human LC dataset reveal an heterogenous and cell-specific expression of YY1 and RKIP in both transformed and non-transformed tissues; Figure S4: Single cell sequencing analyses of deposited LC datasets reveal an heterogenous and cell-specific expression of YY1 and RKIP in both lung normal and lung cancer tissues, with an enrichment of PEBP1 in normal tissues compared to tumor ones.
