# Supplementary material for: Computational Analyses of YY1 and Its Target RKIP Reveal Their Diagnostic and Prognostic Roles in Lung Cancer
Source: Cancers (Basel). 2022 Feb 12;14(4):922. doi: 10.3390/cancers14040922 (PMC8869872; doi:10.3390/cancers14040922)
Supplement: Supplementary file 1 [file cancers-14-00922-s001.zip › cancers-1482174-supplementary.pdf]

Supplementary Materials

Computational Analyses of YY1 and Its Target RKIP Reveal Their Diagnostic and Prognostic Roles in Lung Cancer

Silvia Vivarelli, Luca Falzone, Caterina Maria Grillo, Benjamin Bonavida, Claudia Crimi, Ignazio La Mantia and Massimo Libra

Table S1. YY1-ChIP sequencing results (from 15,000 bp upwards to 5,000 bp downwards the TSS of *RKIP* gene).

Cluster	Cluster Score	Genomic Size	Sample (Experiment ID)	Genome Position	Peak Point	Score	Signal Value	Q-Value (FDR%)
1	330	578	H1-hESC from ENCODE 3 (ENCFF509GYP)	chr12:118120730-118121059	118120895	330	37,797	4,594
1	330	578	K562 from ENCODE 3 (ENCFF024TJO)	chr12:118120782-118121097	118120940	214	22,717	4,195
1	330	578	SK-N-SH from ENCODE 3 (ENCFF363UWP)	chr12:118120754-118121103	118120929	157	23,994	2,834
1	330	578	SK-N-SH from ENCODE 3 (ENCFF363UWP)	chr12:118120958-118121307	118121133	57	8,846	0,128
2	228	330	H1-hESC from ENCODE 3 (ENCFF509GYP)	chr12:118121727-118122056	118121892	227	26,078	4,163
3	492	269	liver from ENCODE 3 (ENCFF459TWF)	chr12:118124490-118124758	118124613	491	79,719	4,772
4	328	376	HepG2 from ENCODE 3 (ENCFF177YDT)	chr12:118126119-118126494	118126307	328	43,354	4,539
5	789	322	liver from ENCODE 3 (ENCFF459TWF)	chr12:118126510-118126831	118126674	788	127,936	4,772
5	789	322	liver from ENCODE 3 (ENCFF838VFX)	chr12:118126554-118126811	118126665	512	74,049	4,760
6	361	584	liver from ENCODE 3 (ENCFF459TWF)	chr12:118131817-118132400	118132109	360	58,536	4,772
7	550	781	A549 from ENCODE 3 (ENCFF613DTQ)	chr12:118135676-118136199	118135938	222	32,101	3,872
7	550	781	GM12878 from ENCODE 3 (ENCFF223MUF)	chr12:118135970-118136313	118136142	238	30,739	5,098
7	550	781	HepG2 from ENCODE 3 (ENCFF177YDT)	chr12:118135832-118136207	118136020	311	41,178	4,539
7	550	781	HepG2 from ENCODE 3 (ENCFF177YDT)	chr12:118136056-118136431	118136244	125	16,556	3,346
7	550	781	liver from ENCODE 3 (ENCFF459TWF)	chr12:118135854-118136064	118135927	550	89,210	4,772
7	550	781	liver from ENCODE 3 (ENCFF838VFX)	chr12:118135651-118136210	118135931	476	68,788	4,760
7	550	781	SK-N-SH from ENCODE 3 (ENCFF363UWP)	chr12:118135809-118136158	118135984	197	30,081	3,680
8	421	631	GM12878 from ENCODE 3 (ENCFF223MUF)	chr12:118136569-118136912	118136741	90	11,628	3,927
8	421	631	GM12878 from ENCODE 3 (ENCFF223MUF)	chr12:118136856-118137199	118137028	50	6,510	1,542
8	421	631	liver from ENCODE 3 (ENCFF459TWF)	chr12:118136589-118137172	118136881	421	68,342	4,772
9	375	798	HepG2 from ENCODE 3 (ENCFF177YDT)	chr12:118139059-118139434	118139247	47	6,223	0,540
9	375	798	liver from ENCODE 3 (ENCFF459TWF)	chr12:118138683-118139266	118138975	374	60,788	4,772
9	375	798	liver from ENCODE 3 (ENCFF459TWF)	chr12:118138897-118139480	118139189	123	20,096	1,850

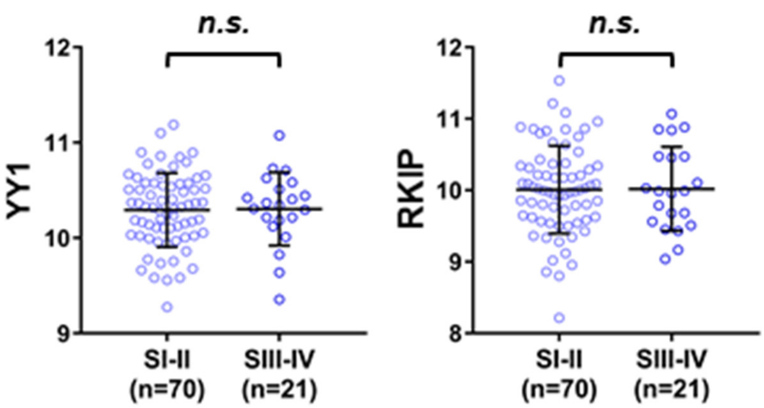

Figure S1. GSE2109, *YY1* (left) and *RKIP* (right) expression in low stage (SI and SII) versus high stage (SIII and SIV) specimens.

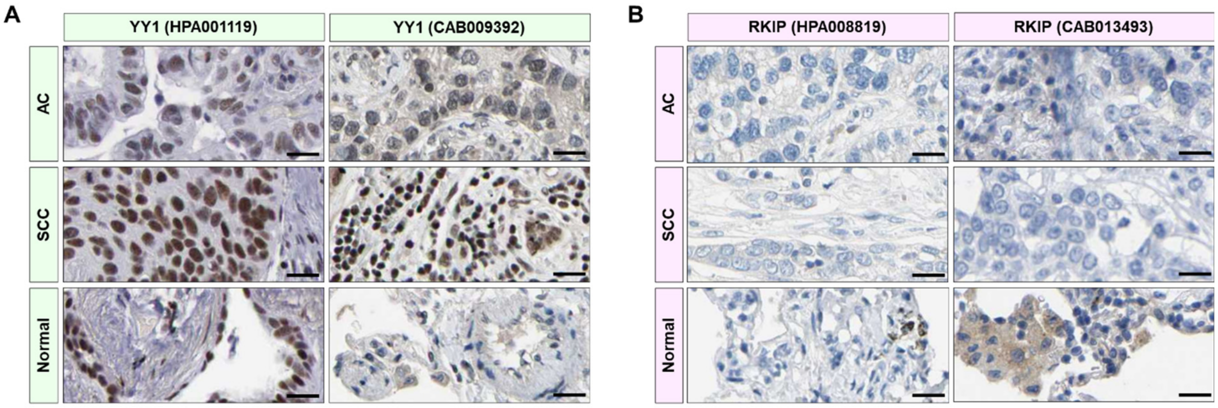

C

	Lung Cancer Samples (n=12)				
	Protein ID	YY1		RKIP	
	antibody ID	*HPA001119*	*CAB009392*	*HPA008819*	*CAB013493*
Tumor #	AC	6	6	6	6
	SCC	4	6	5	6
	n.d.	2	0	0	0
Staining	n.d.	42%	33%	100%	100%
	low	17%	33%		
	medium	42%	33%		
	high				
Intensity	negative	17%	33%	100%	100%
	weak	33%	17%		
	moderate	50%	50%		
	high				
Quantity	0%	17%	33%	100%	100%
	< 25%	33%	17%		
	25%-75%	50%	25%		
	> 75%		25%		
	Localization	N	N	n.d.	n.d.

D

		Normal - Alveolar cells (n=3)				Normal - Macrophages (n=3)			
	Protein ID	YY1		RKIP		YY1		RKIP	
	antibody ID	*HPA001119*	*CAB009392*	*HPA008819*	*CAB013493*	*HPA001119*	*CAB009392*	*HPA008819*	*CAB013493*
Staining	n.d.			100%	100%				
	low							100%	
	medium	100%	100%			100%	100%		100%
	high								
Intensity	negative			100%	100%				
	weak							100%	
	moderate	100%				100%	100%		100%
	high		100%						
Quantity	0%			100%	100%				
	< 25%		100%						
	25%-75%	100%				100%		100%	100%
	> 75%						100%		
	Localization	N	N	n.d.	n.d.	N	N	C, M	C, M

Figure S2. Immunohistochemistry (IHC) analysis of YY1 and RKIP protein expression in Lung Cancer and Normal tissue from the Human Protein Atlas (HPA). A. Representative images of Adenocarcinoma (AC), Squamous Cell Carcinoma (SCC) and Normal (non-transformed) formalin-fixed paraffin-embedded tissues stained with two YY1-specific HPA-validated antibodies (HPA001119 commercialized by Sigma-Aldrich, CAB009392 commercialized by Santa Cruz Biotechnology). Scale bar: 20µm. **B.** Representative images of AC, SCC and Normal tissues stained with two RKIP-specific HPA-validated antibodies (HPA008819 commercialized by Sigma-Aldrich, CAB013493 commercialized by Thermo Scientific). Scale bar: 20µm. **C.** Summary table for Lung cancer samples (n=12): Tumor type, Staining (quality), Intensity (of the staining, averaged), Quantity (of cells stained over the total), Localization (intracellular) of the signal (N, Nuclear; n.d., not determined). **D.** Summary table for Normal samples (n=3): Staining (quality), Intensity (of the staining, averaged), Quantity (of cells stained over the total), Localization (intracellular) of the signal (N, Nuclear; C, Cytoplasmic; M, Membranous; n.d., not determined).

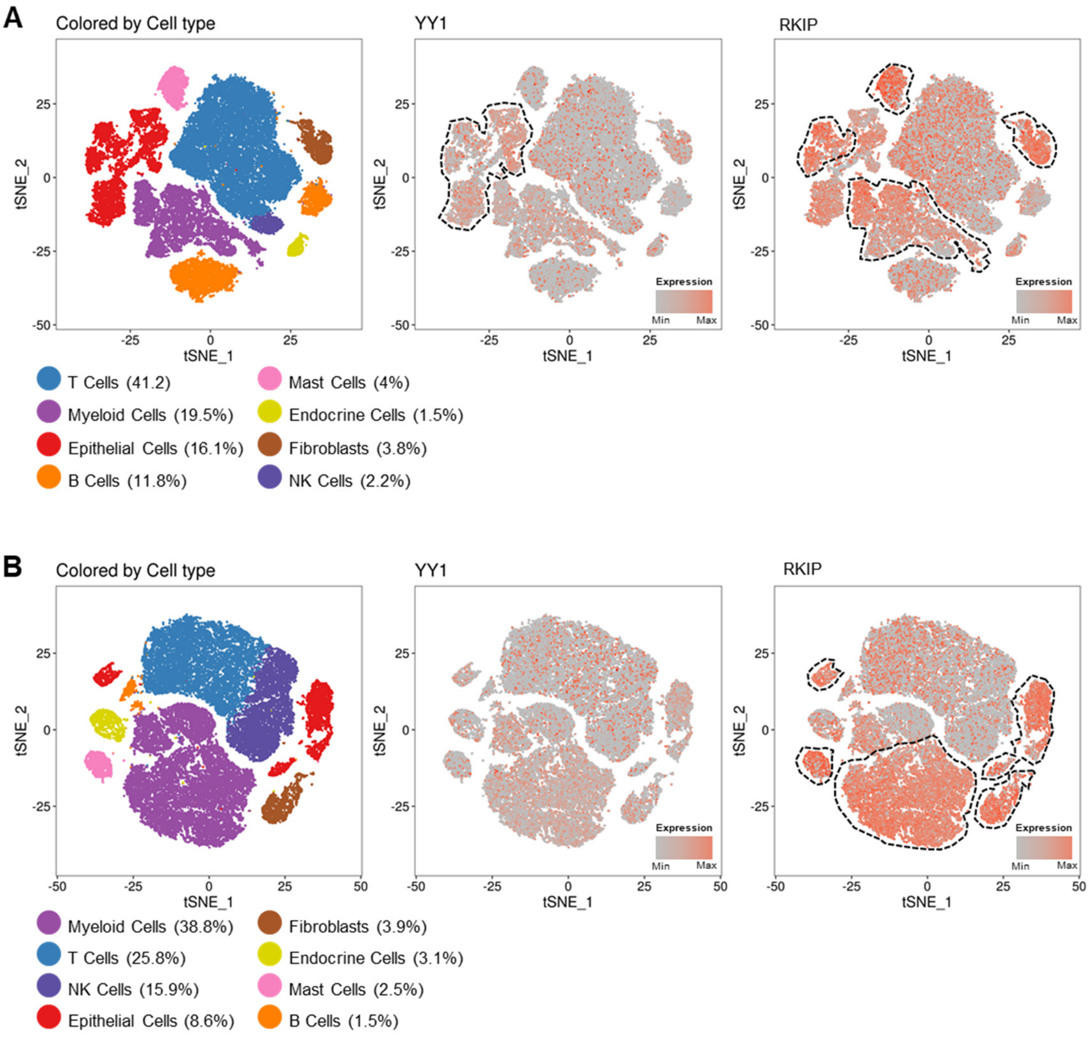

Figure S2. Single cell sequencing analyses of GSE131907 single cell human LC dataset reveal an heterogenous and cell-specific expression of YY1 and RKIP in both transformed and non-transformed tissues. A. t-SNE plots of: cellular clusters distribution, YY1 and RKIP expression distribution in treatment-naïve lung adenocarcinoma cancer patients derived tumor tissue (45,149 cells). Dotted lines evidence clusters and subclusters with YY1 or RKIP high expression. **B.** t-SNE plots of: cellular clusters distribution, YY1 and RKIP expression distribution in treatment-naïve lung adenocarcinoma cancer patients derived non-tumor adjacent tissue (42,995 cells). Dotted clusters and subclusters with RKIP high expression.

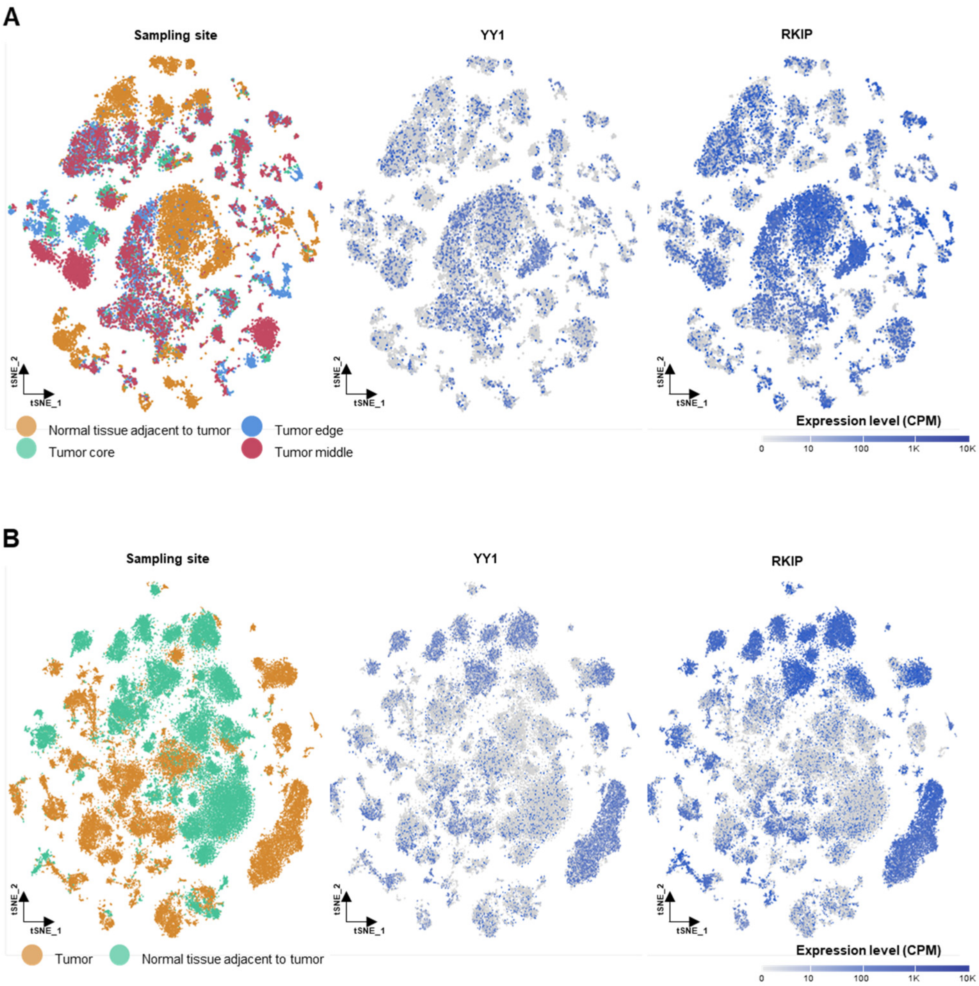

Figure S3. Single cell sequencing analyses of deposited LC datasets reveal an heterogenous and cell-specific expression of *YY1* and *RKIP* in both lung normal and lung cancer tissues, with an enrichment of PEBP1 in normal tissues compared to tumor ones. A. E-MTAB-6653 dataset (52,698 cells). t-SNE plots of cluster populations, *YY1* and *RKIP* gene expression in tumor and normal adjacent cells. **B.** E-MTAB-6308 dataset (56,771 cells), t-SNE plots of cluster populations, *YY1* and *RKIP* gene expression in tumor and normal adjacent cells.
